# Unraveling Let-7f in oncology: a tumor suppressor with emerging clinical significance

**DOI:** 10.3389/fcell.2026.1825514

**Published:** 2026-05-29

**Authors:** Jianhua Deng, Daosheng Li, Zhiqi Li, Yuanming Pan, Xiangsheng Zeng

**Affiliations:** 1 Department of Oncology, Jiujiang City Key Laboratory of Cell Therapy, Jiujiang NO.1 People’s Hospital, Jiujiang, Jiangxi, China; 2 Cancer Research Center, Beijing Chest Hospital, Capital Medical University, Tuberculosis and Thoracic Tumor Research Institute, Beijing, China

**Keywords:** cancer, cancer hallmarks, let-7f, metabolic reprogramming, therapeutic target, tumor microenvironment

## Abstract

As a major global health threat, tumors present significant clinical challenges due to their heterogeneity, metastatic potential, and therapy resistance. In recent years, non-coding RNAs, particularly microRNAs (miRNAs), have emerged as crucial players in cancer research. Among them, let-7f, a key member of the let-7 family, exhibits significant dysregulation and biological functions in various cancers. This review systematically summarizes the differential expression patterns of let-7f in common malignancies, revealing its prevalent downregulation in cancers such as lung, gastric, colorectal, breast, and glioblastoma. This downregulation is closely associated with tumor size, stage, metastatic potential, and patient prognosis. The expression of let-7f is regulated by multiple molecular mechanisms, including transcription factors (e.g., C/EBPβ), RNA-binding proteins (e.g., LIN28), ceRNA networks (e.g., FAM222A-AS1, LINC00106), and genetic polymorphisms. Regarding biological functions, let-7f inhibits tumor cell proliferation, invasion, metastasis, stemness maintenance, and metabolic reprogramming by targeting multiple oncogenes (e.g., MYH9, HMGA2, ADAMTS1, Periostin) and key signaling pathways (e.g., MAPK, Wnt, PI3K/AKT). Furthermore, let-7f is involved in modulating the tumor microenvironment, including angiogenesis, stromal cell function, and the immune milieu. At the therapeutic level, let-7f not only serves as a predictive biomarker for the efficacy of chemotherapy, radiotherapy, and endocrine therapy but also holds potential for reversing drug resistance and enhancing drug sensitivity. For diagnosis, its stable presence in body fluids like plasma and stool offers a non-invasive detection advantage, positioning it as a promising novel biomarker for various cancers. However, challenges remain, including the standardization of detection methods, optimization of delivery systems, and insufficient clinical validation. Future efforts should integrate multi-omics analyses, artificial intelligence-assisted modeling, and novel nanodelivery technologies to advance the translation of let-7f from basic research to clinical application, thereby providing new strategies for the precise diagnosis and treatment of cancer.

## Graphical Abstract

**Figure:**
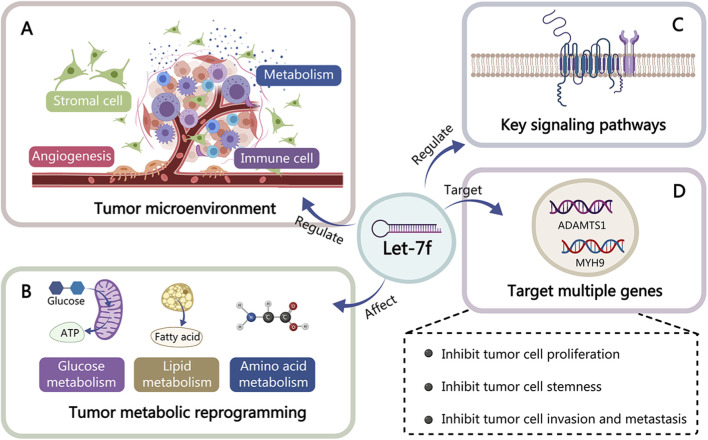
Schematic overview of let-7f functions in the tumor microenvironment, metabolic reprogramming, and cancer hallmark suppression **(A)** Let-7f regulates multiple components of the tumor microenvironment (TME), including stromal cells, angiogenesis, immune cells, and metabolism **(B)** Let-7f is also involved in tumor metabolic reprogramming. It affects glucose metabolism (ATP production), fatty acid/lipid metabolism, and amino acid metabolism, thereby modulating the metabolic adaptation of tumor cells. Through these regulatory actions, let-7f influences key signaling pathways **(C)** and directly targets specific genes such as ADAMTS1 and MYH9 **(D)**, ultimately leading to the inhibition of tumor cell proliferation, stemness, invasion, and metastasis, exerting broad tumor-suppressive effects across various cancer hallmarks.

## 1 Introduction

### 1.1 Background and current status of cancer research

Cancer, a disease posing a severe global threat to human health, has maintained persistently high rates of both incidence and mortality. According to data from the World Health Organization (WHO), there were millions of new incidences as well as the high motability worldwide recently, including dominating lung cancer, breast cancer, colorectal cancer, gastric cancer, and liver cancer. Despite significant advances in recent years regarding the elucidation of molecular mechanisms, optimization of diagnostic technologies, and innovation in treatment strategies, challenges such as tumor heterogeneity, metastatic potential, and treatment resistance continue to severely constrain improvements in clinical efficacy (Siegel et al., 2026; Filho et al., 2025).

In the field of cancer research, the discovery of non-coding RNAs (ncRNAs) has provided a novel perspective for understanding the mechanisms underlying tumorigenesis and progression. Among these, microRNAs (miRNAs), a class of endogenous non-coding RNAs approximately 22 nucleotides in length, regulate gene expression at the post-transcriptional level by binding to the 3'-untranslated region (3′-UTR) of target mRNAs. They are involved in crucial biological processes such as cell proliferation, differentiation, apoptosis, invasion, and metastasis (Xu et al., 2025; Liu et al., 2025). Dysregulation of miRNA expression is closely associated with cancer development. For instance, certain miRNAs function as oncogenes (oncomiRs) to promote tumor progression, while others act as tumor-suppressive miRNAs to inhibit tumorigenesis (Singh et al., 2024). In recent years, a growing body of research has demonstrated that miRNAs can not only serve as biomarkers for cancer diagnosis and prognosis but also hold potential therapeutic value, offering new directions for precision oncology (Reda El Sayed et al., 2021; Michela et al., 2014).

### 1.2 Focusing on let-7f inOncology

The let-7 microRNA family is one of the most extensively studied miRNA families in cancer biology, comprising 13 members across the human genome that encode nine distinct mature miRNAs (Ma et al., 2021; Lee et al., 2016; Roush and Slack, 2008). As a group, let-7 miRNAs function as key tumor suppressors by targeting multiple oncogenes involved in cell cycle regulation, differentiation maintenance, and stemness control (Ma et al., 2021; Balzeau et al., 2017; Yazarlou et al., 2021; Zhang et al., 2021; Xiao et al., 2023). However, while the let-7 family shares a conserved seed sequence and generally exerts tumor-suppressive functions, emerging evidence reveals that individual family members exhibit distinct expression patterns, regulatory mechanisms, and clinical associations across different cancer types. The present manuscript specifically focuses on let-7f for the following reasons.

First, let-7f demonstrates unique functional specificity in cancer regulation. Although all let-7 members share common target genes, let-7f has been identified as a critical player in several cancer hallmarks that are less prominently regulated by other family members. For instance, let-7f directly targets β2-adrenergic receptor (β2-AR) in breast cancer, a regulatory axis that is not a major function of other let-7 members (Liu Dan et al., 2015). MEK1/2 inhibitors PD98059 or PD184352 significantly upregulates let-7f expression, and let-7f in turn suppresses β2-AR, establishing a negative feedback loop that associates with lymph node metastasis and poor outcome in the patients with Her2-positive breast cancer. This endocrine-specific regulatory role positions let-7f as a uniquely valuable biomarker and therapeutic target in hormone-responsive cancers.

Second, let-7f exhibits distinct expression profiles and clinical correlations compared to other let-7 members. In uterine leiomyosarcoma, a comprehensive profiling study that examined eight let-7 family members (let-7a through let-7i) found that while all members were downregulated in tumor tissues, only let-7e independently predicted overall survival, whereas let-7b and let-7d influenced disease-free survival; notably, let-7f downregulation was significantly associated with patient age, with the lowest expression levels observed in the oldest patients (de Almeida et al., 2019). These differential patterns underscore that let-7f is not merely a surrogate for the entire let-7 family but carries distinct prognostic implications that merit focused investigation.

Third, let-7f is uniquely positioned at the interface of multiple cancer hallmarks and the tumor microenvironment. As demonstrated throughout this manuscript, let-7f regulates tumor cell proliferation via targeting ADAMTS1, AKT2, HMGA2, and FZD3 (Zafari et al., 2022; Wen et al., 2020; Li et al., 2016); suppresses invasion and metastasis through MYH9, integrin β1, POSTN, and β2-AR (Liu Dan et al., 2015; Liang et al., 2011; Yan et al., 2015; Yang et al., 2021; Xue et al., 2016); modulates stemness via HMGA2 and MYH9 (Zafari et al., 2022; Song et al., 2022); participates in metabolic reprogramming through c-Myc and HK2 (Yang Ting et al., 2020; Chien-Hsiu and Chiao-Chun, 2021); and mediates exosomal crosstalk between mesenchymal stem cells and tumor cells within the TME (Egea et al., 2021). This multifaceted regulatory capacity—spanning proliferation, metastasis, stemness, metabolism, and microenvironmental interactions—is not equivalently represented by other let-7 members, making let-7f a particularly compelling candidate for integrated analysis of tumor hallmarks within the TME framework.

Fourth, accumulating evidence positions let-7f as a promising translational target with specific therapeutic and diagnostic applications. In gastric cancer, let-7f is a promising therapeutic candidate for gastric cancer to reduce cell invasion and metastasis by targeting MYH9 (Liang et al., 2011). In non-small cell lung cancer, let-7f overexpression suppresses proliferation, migration, and invasion by targeting TGFBR1 (Yang et al., 2021). In leukemia, let-7f reverses multidrug resistance by targeting ABCC5 and ABCC10 (Cao et al., 2020). In colorectal cancer, let-7f-5p is significantly downregulated in plasma and stool, offering a non-invasive diagnostic approach (Ghanbari et al., 2015). In prostate cancer, plasma let-7f-5p combined with PSA significantly improves diagnostic accuracy over PSA alone (Ge et al., 2020). These clinical and preclinical observations highlight that let-7f has advanced further toward translational applications than many other family members.

Collectively, while all let-7 family members contribute to tumor suppression, let-7f possesses distinctive functional attributes—including its role in endocrine therapy regulation, its specific target repertoire, its association with age-related expression changes, its broad impact across multiple cancer hallmarks within the TME, and its emerging diagnostic and therapeutic potential—that justify its focused investigation. Understanding these family-specific differences is essential for developing precision medicine strategies that leverage the unique biological properties of individual let-7 members rather than treating the family as a functionally redundant group.

### 1.3 Purpose and structure of the review

Although the let-7 family is well recognized for its tumor-suppressive functions, emerging evidence indicates that individual family members possess distinct biological properties and clinical relevance. Among them, let-7f stands out due to its unique capacity to simultaneously regulate multiple cancer hallmarks—including proliferation, invasion, stemness, and metabolic reprogramming—within the context of the tumor microenvironment (TME). Moreover, let-7f exhibits specific regulatory axes not shared by other let-7 members, such as its direct targeting of the aromatase gene *CYP19A1* in hormone-responsive cancers, and its active involvement in exosome-mediated crosstalk between mesenchymal stem cells and tumor cells (Shibahara et al., 2012). These distinctive features justify a dedicated, systematic review focused on let-7f rather than on the let-7 family as a whole.

Nevertheless, a comprehensive synthesis of let-7f expression patterns, regulatory networks, and clinical applications across different cancer types is still lacking. Therefore, this review aims to provide an integrated overview of the differential expression, biological functions, mechanisms of action, and clinical prospects of let-7f in oncology. By doing so, we hope to offer a valuable reference for future mechanistic investigations and to inspire new diagnostic and therapeutic strategies centered on let-7f.

The review is structured as follows: Part 2 details the expression characteristics of let-7f in common cancers, including its altered expression levels across tumor types, correlations with clinicopathological features, and the molecular mechanisms underlying its dysregulation. Part 3 systematically explores the roles of let-7f in tumorigenesis and progression, with emphasis on its regulation of tumor cell proliferation, invasion and metastasis, the tumor microenvironment, cancer stemness, and metabolic reprogramming. Part 4 analyzes the relationship between let-7f and cancer therapy, covering its potential as a therapeutic target, its impact on treatment efficacy, and its association with therapy resistance. Part 5 summarizes the clinical applications and future perspectives of let-7f, including its diagnostic biomarker potential, exploratory therapeutic applications, and the challenges and opportunities for clinical translation.

Through this systematic review, we aim to present a comprehensive and up-to-date understanding of the research progress and translational value of let-7f in oncology.

## 2 Differential expression of let-7f in tumors

### 2.1 Expression characteristics of let-7f in common tumors

As a crucial member of the let-7 family, the expression pattern of let-7f in tumors exhibits significant tissue specificity and disease stage dependency, with most studies supporting its core function as a tumor suppressor (Table 1). In central nervous system tumors, differential expression of let-7f-5p can be used to distinguish primary CNS lymphoma (PCNSL) from glioblastoma (GBM). Sequencing of plasma exosomal miRNAs and subsequent RT-qPCR validation in 27 PCNSL and 27 GBM patients revealed that let-7f-5p expression was significantly higher in the PCNSL group than in the GBM group (p = 0.036), a difference potentially attributable to variances in cellular origin and molecular regulatory networks between the two tumor types (Lu et al., 2025). In the field of lung cancer, downregulation of let-7f expression is a common feature across various subtypes. The expression of let-7f-5p in the plasma of lung adenocarcinoma patients is significantly lower than in healthy controls, and its low expression is associated with malignant phenotypes in tumor epithelial cells (Faversani et al., 2021). Similarly, plasma exosomal let-7f-5p levels are markedly reduced in NSCLC patients (p < 0.0001), with even lower expression observed in metastatic NSCLC patients compared to non-metastatic ones (p = 0.023), suggesting an inverse correlation between its expression level and tumor invasive capacity (Wang et al., 2020). Furthermore, downregulated let-7f-5p expression in NSCLC tissues is directly linked to the overexpression of oncogenes such as HMGA2 and FZD3, further validating its tumor-suppressive role (Zafari et al., 2022).

**TABLE 1:** Summary of Differential Expression of let-7f in Tumors.

Tumor type	Common expression pattern	Clinical significance
Non-small cell lung cancer (Silva et al., 2010)	Decreased	Related to overall survival and poor prognosis
Colorectal cancer (Ghanbari et al., 2015; Bakhsh et al., 2024; Tie et al., 2018)	Differential expression	Related to the occurrence and development of tumor cells, upregulation can promote chemoresistance in CRC
Hepatocellular carcinoma (Ge et al., 2014)	Decreased	Related to the biological behavior of HCC
Gastric cancer (Liang et al., 2011)	Decreased	Inhibit the invasion and migration
Breast cancer (Liu et al., 2015a; Mattie et al., 2006; Turkistani et al., 2021)	Decreased	Related to breast cancer metastasis and poor prognosis, associated with the aggressiveness of HER2-positive or triple-negative breast cancer
Ovarian cancer (Zheng et al., 2013)	Decreased	Related to the metastasis and poor prognosis of ovarian cancer
Prostate cancer (Kang et al., 2017)	Decreased	promoted the growth, clonal proliferation, and invasiveness of tumor cells
Endometrial cancer (Jayaraman et al., 2017; Lazaridis et al., 2025)	Decreased	Promote the growth and recurrence of endometrial cancer
Acute myeloid leukemia (Cao et al., 2020; Dai et al., 2014)	Decreased	Mediate chemotherapy resistance in leukemia
Glioblastoma (Matos et al., 2018)	Decreased	Related to prognosis and overall survival rate

In oral tumors, the expression pattern of let-7f is closely associated with tumor aggressiveness. Comparative analysis of tumor tissues versus normal mucosa from 12 young oral tongue squamous cell carcinoma patients under 30 years old showed an overall upregulation trend of let-7f-5p in tumor tissues. However, its expression was significantly higher in non-invasive tumors compared to invasive ones, suggesting a potential dose-dependent regulatory mechanism in tumor progression within this young patient cohort (Hilly et al., 2016).

In digestive system tumors, aberrant expression of let-7f is evident throughout disease development. Patients with colorectal cancer (CRC) show significantly low expression of let-7f-5p in both plasma and stool samples. Notably, the expression level of let-7f in stool demonstrates high diagnostic sensitivity and specificity for early-stage CRC (Ghanbari et al., 2015). Low let-7f expression in CRC tissues negatively correlates with high expression of FAM222A-AS1, which sponges let-7f via a ceRNA mechanism, consequently upregulating pro-metastatic genes like MYH9 (Song et al., 2022). In gastric cancer, let-7f expression is significantly lower in cell lines with high metastatic potential (e.g., GC9811-P, SGC7901-M) compared to their parental lines. Let-7f can directly target MYH9 to inhibit the invasion and migration of gastric cancer cells (Liang et al., 2011). Conversely, serum let-7f is highly expressed in gastric cancer patients and is associated with the progression from precancerous lesions (atrophic gastritis) to gastric cancer (Liu WJ. et al., 2015). In pancreatic cancer, fine-needle aspiration (FNA) specimens show significantly reduced let-7f expression, which, together with other tumor-suppressive miRNAs like miR-200c, contributes to the molecular signature of this malignancy (Ali et al., 2012).

In endocrine system tumors, aberrant expression of let-7f is involved in malignant transformation. In patients with papillary thyroid carcinoma (PTC), plasma let-7f expression is significantly higher than in healthy controls. RET/PTC3 oncogene activation can markedly reduce let-7f expression in PCCL3 rat thyroid cells, while let-7f overexpression inhibits MAPK pathway activation in TPC-1 cells, reduces cell proliferation, and promotes the expression of thyroid differentiation markers (e.g., TITF1, TG) (Perdas et al., 2020; Ricarte-Filho et al., 2009). However, in aggressive PTC patients with the BRAF T1799A mutation, let-7f expression is low, suggesting its expression pattern may be influenced by tumor driver gene mutations (Geraldo et al., 2012).

In renal cell carcinoma (RCC), serum let-7f-5p expression in patients with clear cell RCC (ccRCC) is significantly lower than in healthy controls. A diagnostic panel comprising let-7f-5p, miR-27b-3p, and miR-142-5p demonstrates a high AUC value (0.952) (He et al., 2024). In metastatic RCC cells, the high expression of ADAMTS1 can be suppressed by melatonin through the induction of let-7f and other miRNAs, thereby reducing tumor invasiveness (Wen et al., 2020).

In reproductive system tumors, low let-7f expression is associated with poor prognosis. In uterine leiomyosarcoma (LMS) patients, let-7f is significantly downregulated in tumor tissues, and this low expression correlates with patient age, with lower levels observed in older patients (de Almeida et al., 2019). In ovarian cancer, highly invasive cell lines (e.g., SKOV-3ip, HO-8910PM) exhibit significantly lower let-7f expression compared to less invasive lines (e.g., SKOV-3, HO-8910), suggesting a potential role in inhibiting metastasis-related pathways (Liang et al., 2010). Reduced plasma let-7f expression can serve as a diagnostic biomarker for ovarian cancer and is associated with poor patient prognosis (Zheng et al., 2013).

In breast tumors, let-7f expression is regulated by multiple factors. HER2-overexpressing breast cancer cells show significantly decreased let-7f expression, a process dependent on ERK pathway activation. Conversely, let-7f overexpression can inhibit tumor progression by targeting β2-AR (Liu Dan et al., 2015). Low-dose metronomic paclitaxel treatment reduces let-7f expression in breast cancer tissues, subsequently upregulating the anti-angiogenic factor TSP-1 (Wei-Yang et al., 2015). Furthermore, let-7f expression in breast tumors negatively correlates with LIN28, which promotes tumorigenesis by inhibiting let-7f maturation (Sakurai et al., 2011).

In tumors of other systems, aberrant expression of let-7f also holds significant importance. In hepatocellular carcinoma (HCC), serum let-7f expression is decreased in patients, and its low expression is associated with larger tumor size (>5 cm) and early recurrence (Ge et al., 2014). LINC00106, upregulated through m6A methylation modification, promotes stemness and metastasis in HCC cells by sponging let-7f (Liang et al., 2021). In patients with HCV-related HCC, serum let-7f-1 expression is significantly downregulated and, together with miRNAs like miR-143/145, contributes to hepatocarcinogenesis (Aly et al., 2020).

In leukemia, the multidrug-resistant cell line K562/A02 exhibits significantly lower let-7f expression compared to the sensitive parental line K562. This low expression mediates doxorubicin resistance by upregulating drug-resistance genes such as ABCC5 and ABCC10 (Cao et al., 2020).

In neuroendocrine tumors, low expression of let-7f family members can lead to the upregulation of target genes like EGR1 and G3BP1, thereby promoting tumor growth and metastasis (Døssing et al., 2014).

### 2.2 Molecular regulatory mechanisms underlying differential expression

**FIGURE 1:**
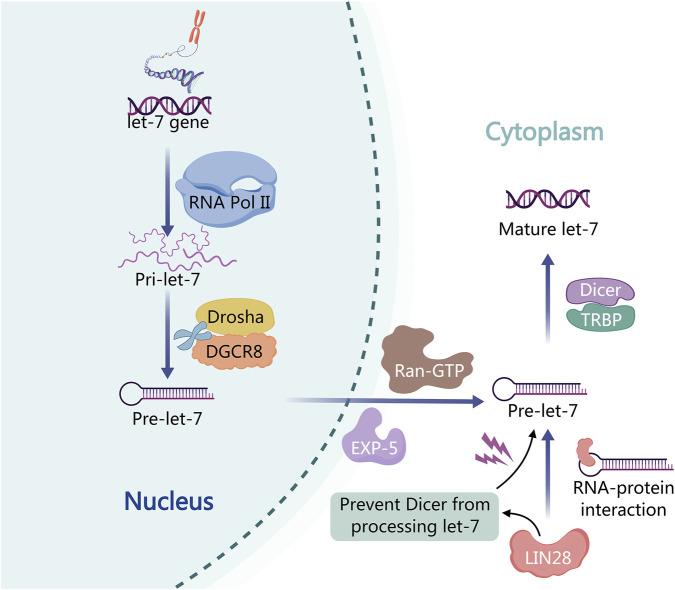
Schematic illustration of let-7 biogenesis. The let-7 gene is transcribed by RNA polymerase II (RNA Pol II) to generate the primary transcript, pri-let-7. In the nucleus, pri-let-7 is cleaved by the microprocessor complex consisting of Drosha and DGCR8 to produce precursor let-7 (pre-let-7). Pre-let-7 is then exported from the nucleus to the cytoplasm via Exportin 5 (EXP-5) in a Ran-GTP-dependent manner. Once in the cytoplasm, pre-let-7 is further processed by the Dicer/TRBP complex to generate mature let-7, which is subsequently loaded into RNA-protein complexes (e.g., RNA-induced silencing complex, RISC) to exert its regulatory functions. LIN28 negatively regulates let-7 biogenesis by binding to pre-let-7, thereby preventing Dicer from accessing and processing the precursor.

The differential expression of let-7f is regulated at multiple levels, including transcriptional control, post-transcriptional modifications, and epigenetic mechanisms. Core regulatory pathways involve transcription factor binding, RNA-binding protein modulation, competing endogenous RNA (ceRNA) networks, and genetic polymorphisms (Figure 1).

At the transcriptional level, CCAAT/enhancer-binding protein β (C/EBPβ) serves as a key transcriptional activator for let-7f-1. In human endocervical epithelial cells (End1/E6E7), the let-7f-1 promoter region contains six potential C/EBPβ binding sites. Knockdown of C/EBPβ significantly reduces let-7f expression, while its overexpression promotes let-7f expression. Treatment with the TLR-3 ligand poly (I:C) enhances the binding of C/EBPβ to sites 3, 5, and 6 within the promoter, indicating that let-7f expression is modulated by immune signaling pathways (Kanchana and Reddy, 2017). Furthermore, the MAPK and NF-κB signaling pathways jointly participate in the transcriptional regulation of let-7f. In End1/E6E7 cells, TLR3 stimulation activates the p38, JNK, ERK, and NF-κB pathways. Activation of the p38 and JNK pathways directly promotes let-7f expression, while inhibition of the ERK and NF-κB pathways indirectly enhances let-7f expression by upregulating C/EBPβ (Kanchana and Reddy, 2018).

At the post-transcriptional level, LIN28 family proteins are critical negative regulators of let-7f maturation. In breast cancer cells, LIN28 expression shows a significant negative correlation with let-7f levels. LIN28 binds to the precursor of let-7f (pre-let-7f), inhibiting its processing into the mature miRNA, thereby alleviating the suppression of downstream oncogenes (Sakurai et al., 2011). In End1/E6E7 cells, changes in C/EBPβ expression inversely regulate the expression of LIN28 A/B, suggesting the existence of a “C/EBPβ–LIN28–let-7f” feedback regulatory loop (Kanchana and Reddy, 2017).

The ceRNA mechanism represents a significant mode of post-transcriptional regulation for let-7f. Long non-coding RNAs (lncRNAs) can function as molecular sponges to sequester let-7f, thereby modulating its activity. In colorectal cancer, FAM222A-AS1 is highly expressed in tumor tissues. Containing let-7f binding sites in its sequence, it directly binds to and inhibits let-7f activity, leading to the upregulation of MYH9 expression and the promotion of tumor cell proliferation and migration (Song et al., 2022). In hepatocellular carcinoma, LINC00106 undergoes m6A modification mediated by m6A methyltransferases, enhancing its stability. This stabilized LINC00106 then acts as a sponge for let-7f, resulting in the upregulation of periostin expression, activation of the PI3K-AKT pathway, and the promotion of tumor stemness and metastasis (Liang et al., 2021).

Additionally, exosome-mediated transport of let-7f contributes to the regulation of the tumor microenvironment. Human bone marrow-derived mesenchymal stem cells (hMSCs) upregulate let-7f expression under stimulation by SDF-1α or hypoxic conditions. They subsequently package let-7f into exosomes for delivery to breast cancer cells, where it inhibits tumor growth (Egea et al., 2021). Exosomes secreted by GATA-4-overexpressing MSCs contain elevated levels of let-7f, which can promote angiogenesis by downregulating THBS1 expression in endothelial cells (Gong et al., 2022).

Genetic polymorphisms significantly influence let-7f expression. In colorectal cancer patients, the AG/AA genotype at the rs17276588 locus in pri-let-7f-2 is associated with significantly lower transcriptional activity of let-7f compared to the GG genotype. Consequently, tumor tissues from carriers of the AG/AA genotype exhibit lower let-7f expression levels. Moreover, patients harboring the AG/AA genotype have a significantly increased risk of CRC (adjusted OR = 1.43, 95% CI = 1.17–1.75, p < 0.001). This suggests that this polymorphism contributes to tumorigenesis by affecting let-7f expression (Yuan et al., 2019).

Furthermore, environmental factors such as HCV infection can influence let-7f expression via epigenetic modifications. The downregulation of serum let-7f-1 in patients with HCV-related HCC may be associated with HCV-induced alterations in DNA methylation or histone modifications (Aly et al., 2020).

### 2.3 Association between differential expression and pathological features in tumors

The differential expression of let-7f is closely associated with key pathological features of tumors, including pathological grade, clinical stage, metastatic potential, and prognosis. Its expression level can serve as an important indicator for evaluating tumor aggressiveness and predicting patient outcomes.

Regarding tumor size, the serum expression level of let-7f in HCC patients significantly correlates with tumor size. Patients with tumors larger than 5 cm in diameter exhibit significantly higher let-7f expression compared to those with tumors ≤5 cm (p = 0.0367), suggesting that let-7f may influence tumor growth by regulating cell proliferation-related pathways (Ge et al., 2014). In CRC, patients carrying the AG/AA genotype at the rs17276588 locus tend to have better tumor differentiation (moderately/well-differentiated) and are predominantly diagnosed at earlier clinical stages (I-II). This implies that low let-7f expression might confer a growth advantage in the early phases of tumor development (Yuan et al., 2019).

In terms of clinical stage and metastatic potential, let-7f expression levels show an inverse correlation with tumor invasiveness and metastatic capacity. In NSCLC patients, plasma exosomal let-7f-5p expression is significantly lower in those with metastatic disease compared to non-metastatic patients (p = 0.023). Furthermore, the combination of let-7f with miR-320a and miR-622 can effectively distinguish between metastatic and non-metastatic NSCLC cases (Wang et al., 2020). In ovarian cancer, decreased plasma let-7f expression is associated with advanced-stage disease (III-IV), and its low levels significantly correlate with poor patient prognosis (Zheng et al., 2013). In gastric cancer, cell lines with high metastatic potential show significantly lower let-7f expression than those with low metastatic potential. Overexpression of let-7f can inhibit the migration and invasion of gastric cancer cells by targeting MYH9 (Liang et al., 2011). Additionally, in oral tongue squamous cell carcinoma, let-7f-5p expression is significantly higher in non-invasive tumors compared to invasive ones, further validating its role as a metastasis suppressor (Hilly et al., 2016).

In terms of prognostic evaluation, let-7f expression levels can serve as an independent prognostic factor for various tumors. In HCC patients, decreased serum let-7f expression is significantly associated with early tumor recurrence (p = 0.0047), suggesting its potential use in predicting recurrence risk (Ge et al., 2014). In NSCLC patients, plasma exosomal let-7f levels correlate with overall survival, with lower expression linked to significantly reduced survival rates (Silva et al., 2010). In ovarian cancer patients, low plasma let-7f expression is associated with poor prognosis and can serve as an independent prognostic predictor (Zheng et al., 2013). Furthermore, in CRC patients, the combination of low let-7f expression and high FAM222A-AS1 expression indicates a poor prognosis, and the FAM222A-AS1/let-7f/MYH9 pathway represents a potential therapeutic target (Song et al., 2022).

Regarding pathological subtypes, differential expression of let-7f can help distinguish between different tumor types. Significant differences in plasma exosomal let-7f-5p expression exist between primary central nervous system lymphoma (PCNSL) and GBM, highlighting its potential as a diagnostic biomarker for differentiating these two malignancies (Lu et al., 2025). In breast cancer, patients with neuroendocrine features (BC-NEFs) exhibit significantly lower let-7f expression compared to those with invasive ductal carcinoma (IDC) without neuroendocrine features, suggesting a potential role for let-7f in neuroendocrine differentiation (Usul et al., 2024). In pancreatic cancer, low let-7f expression is associated with the pathological features of pancreatic adenocarcinoma and may serve as one of its molecular diagnostic markers (Ali et al., 2012).

Concerning treatment response, let-7f expression levels can predict tumor sensitivity to therapy. In ovarian cancer, let-7f expression correlates with the response to carboplatin/paclitaxel chemotherapy, with patients exhibiting high let-7f expression showing greater chemosensitivity (García-Vázquez et al., 2018). Low-dose metronomic paclitaxel treatment reduces let-7f expression in breast cancer tissues, subsequently upregulating TSP-1 and enhancing anti-angiogenic effects (Ge et al., 2020). Additionally, melatonin can enhance tumor sensitivity to treatment by inducing let-7f and other miRNAs to suppress ADAMTS1 expression in metastatic renal cell carcinoma (Wen et al., 2020).

In summary, the differential expression of let-7f across various tumor types exhibits high tissue specificity and pathological relevance. Its regulation involves multiple levels, including transcriptional, post-transcriptional, and epigenetic mechanisms. Furthermore, its expression level is closely linked to key pathological features, metastatic potential, and prognosis. In-depth investigation into the expression patterns and regulatory mechanisms of let-7f may provide new targets and strategies for tumor diagnosis, treatment, and prognostic assessment.

## 3 Let-7f in the tumor microenvironment: Regulation of cancer hallmarks

The tumor microenvironment (TME) is a complex ecosystem comprising tumor cells, stromal cells, immune cells, extracellular matrix, and signaling molecules. The TME not only supports tumor growth but also drives key cancer hallmarks such as sustained proliferation, invasion, metastasis, stemness, and metabolic reprogramming. As a tumor-suppressive miRNA, let-7f acts within the TME to regulate multiple hallmarks. This section integrates the functions of let-7f across different cancer hallmarks into the context of TME regulation.

### 3.1 Let-7f regulates tumor angiogenesis and metabolic reprogramming in the TME

Angiogenesis and metabolic reprogramming are two interconnected hallmarks that enable tumor cells to survive and proliferate within the TME. let-7f plays a dual role in these processes depending on cancer type.

In angiogenesis, let-7f exhibits context-dependent functions. In diffuse large B-cell lymphoma (DLBCL), let-7f acts as a pro-angiogenic miRNA (angiomiR); its elevated expression correlates with microvessel density, promoting tumor growth by enhancing nutrient and oxygen supply (Borges et al., 2016). Conversely, in breast cancer, let-7f exerts anti-angiogenic effects by directly targeting the 3′-UTR of thrombospondin-1 (TSP-1), an inhibitor of angiogenesis. Low-dose metronomic paclitaxel reduces let-7f expression, thereby upregulating TSP-1 to inhibit angiogenesis (Wei-Yang et al., 2015). In glioma, let-7f targets periostin (POSTN) to suppress vascular mimicry—a process where tumor cells form vessel-like structures—thus limiting blood supply (Yan et al., 2015; Xue et al., 2016).

In metabolic reprogramming, let-7 family members, including let-7f, regulate glycolysis, glutamine metabolism, and fatty acid synthesis. let-7 can directly target key glycolytic enzymes such as hexokinase 2 (HK2) and PFKFB3, as well as transcription factors like c-Myc, thereby reducing glycolytic flux (Chien-Hsiu and Chiao-Chun, 2021). In colorectal cancer (CRC), the lncRNA MAPKAPK5-AS1 sponges let-7f-1-3p, leading to increased c-Myc expression and indirectly promoting glycolysis (Yang Ting et al., 2020). In osteosarcoma, the let-7f-5p/TARBP2 feedback loop is suppressed under hypoxia, activating the Wnt pathway and promoting autophagy-mediated metabolic adaptation (Chen et al., 2020). In prostate cancer, lycopene upregulates let-7f-1, which inhibits AKT2, a key regulator of the PI3K/AKT/mTOR metabolic pathway (Li et al., 2016). In NSCLC, let-7f-5p targets HMGA2 to suppress metabolic reprogramming (Zafari et al., 2022).

Within the TME, let-7f modulates angiogenesis (either pro- or anti-angiogenic depending on context) and suppresses metabolic reprogramming by targeting glycolysis- and lipid synthesis-related genes. These actions collectively limit tumor growth and adaptation to microenvironmental stress.

### 3.2 Let-7f modulates tumor invasion, metastasis, and stemness via TME components

Invasion, metastasis, and stemness are hallmarks closely linked to TME dynamics, including extracellular matrix (ECM) remodeling, epithelial-mesenchymal transition (EMT), and cancer stem cell (CSC) niches. let-7f suppresses these processes through multiple targets.

**FIGURE 2:**
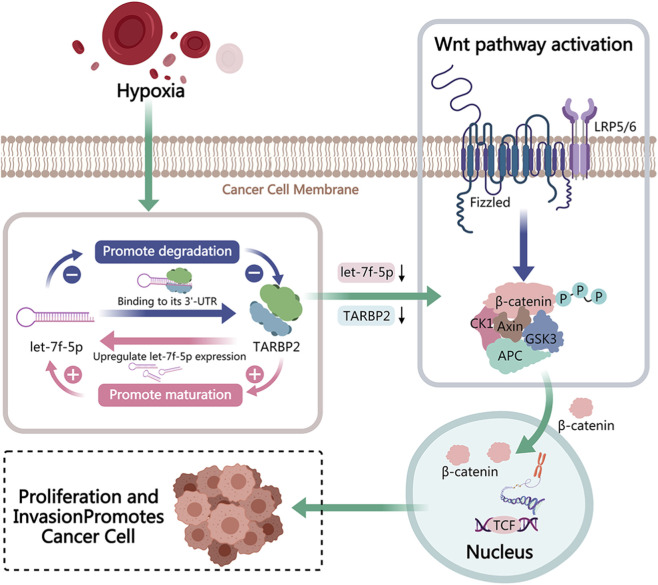
The let-7f-5p/TARBP2 feedback loop regulates Wnt signaling and promotes cancer cell proliferation and invasion under hypoxia. Under hypoxic conditions, the expression of let-7f-5p and TARBP2 is dysregulated. TARBP2 promotes the maturation of let-7f-5p, thereby upregulating its expression. In turn, let-7f-5p binds to the 3′-UTR of TARBP2 mRNA, promoting its degradation and forming a negative feedback loop. Hypoxia-induced suppression of this loop leads to activation of the Wnt signaling pathway: Wnt ligands bind to Frizzled (Fzd) and LRP5/6 co-receptors, stabilizing β-catenin, which then translocates to the nucleus to drive transcription of target genes involved in proliferation and invasion. Consequently, disruption of the let-7f-5p/TARBP2 feedback loop under hypoxic conditions promotes cancer cell proliferation and invasion.

Invasion and metastasis: In renal cell carcinoma (RCC), let-7f targets ADAMTS1; melatonin induces let-7f to reduce ADAMTS1 expression, thereby inhibiting invasion (Wen et al., 2020). In gastric cancer, let-7f directly targets MYH9 (encoding myosin IIA), suppressing invasion and lung metastasis (Liang et al., 2011). In CRC, lncRNA MAPKAPK5-AS1 sponges let-7f-1-3p, leading to SNAI1 upregulation and EMT promotion (Yang Ting et al., 2020); additionally, let-7f-5p negatively correlates with perineural invasion via IGF axis regulation (Niculae et al., 2022). In NSCLC, let-7f-1-3p targets integrin β1, reducing migration and invasion (Yang et al., 2021). In ovarian cancer, low let-7f expression correlates with high invasiveness (Zheng et al., 2013; Liang et al., 2010). In glioma, let-7f targets POSTN to inhibit migration and invasion (Yan et al., 2015; Xue et al., 2016). In breast cancer, let-7f directly targets β2-adrenergic receptor (β2-AR); HER2 overexpression suppresses let-7f, leading to β2-AR upregulation and lymph node metastasis (Ghanbari et al., 2015). In osteosarcoma, hypoxia-induced downregulation of the let-7f-5p/TARBP2 loop activates Wnt signaling, promoting invasion (Chen et al., 2020) (Figure 2).

Stemness: let-7f reduces cancer stem cell properties by targeting stemness-associated genes. In thyroid cancer, restoring let-7f suppresses MAPK signaling and promotes differentiation (Ricarte-Filho et al., 2009). In breast cancer, Lin28 inhibits let-7 maturation, leading to increased HMGA2 and c-Myc, thereby enhancing stemness (Sakurai et al., 2011). In NSCLC, let-7f-5p targets HMGA2 to reduce stemness (Zafari et al., 2022). In glioma, let-7f targets POSTN, downregulating stemness markers CD133 and Nestin (Yan et al., 2015; Xue et al., 2016). In CRC, the FAM222A-AS1/let-7f/MYH9 axis regulates stemness markers CD44 and ALDH1 (Song et al., 2022). In osteosarcoma, the let-7f-5p/TARBP2 loop under hypoxia activates Wnt and promotes stemness markers SOX2/OCT4 (Chen et al., 2020) (Figure 2). In prostate cancer, lycopene-induced let-7f-1 inhibits AKT2 and reduces CD44/ALDH1 expression (Li et al., 2016).

By targeting ECM-related molecules (ADAMTS1, POSTN, integrin β1), cytoskeletal regulators (MYH9), EMT transcription factors (SNAI1), and stemness regulators (HMGA2, AKT2), let-7f suppresses invasion, metastasis, and stemness within the TME, thereby counteracting these aggressive cancer hallmarks.

### 3.3 Let-7f mediates crosstalk between tumor cells and stromal/immune cells in the TME

Beyond direct effects on tumor cells, let-7f regulates interactions with stromal and immune cells, shaping the TME.

**FIGURE 3:**
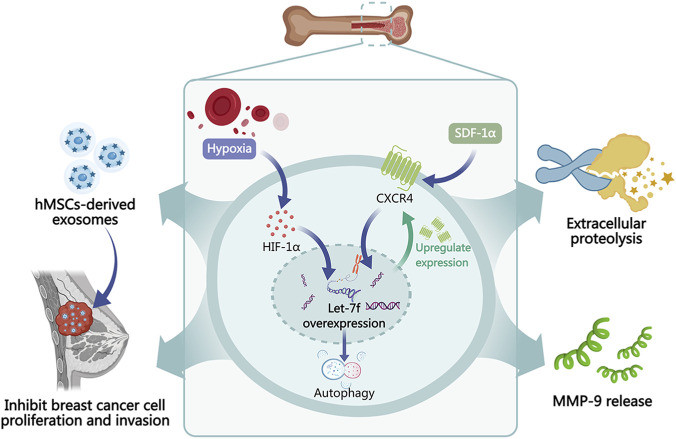
Schematic diagram illustrating the mechanism by which let-7f overexpression inhibits breast cancer cell proliferation and invasion. Under hypoxic conditions or upon stimulation with SDF-1α, let-7f expression is upregulated. This overexpression of let-7f activates autophagy, promotes extracellular proteolysis, and enhances the release of matrix metalloproteinase-9 (MMP-9). These coordinated events ultimately lead to the inhibition of breast cancer cell proliferation and invasion. The involvement of CXCR4 (the receptor for SDF-1α) and HIF-1α (a key transcription factor induced by hypoxia) is indicated as upstream regulators of let-7f upregulation.

Stromal cells (mesenchymal stem cells, MSCs): In human MSCs (hMSCs), SDF-1α or hypoxia upregulates let-7f, enhancing CXCR4-dependent invasion via increased MMP-9 release. Importantly, let-7f is loaded into exosomes secreted by hMSCs under these conditions; these exosomes are taken up by breast cancer cells (4T1), where they inhibit proliferation and invasion (Egea et al., 2021) (Figure 3). Additionally, TIMP-1 suppresses let-7f in hMSCs, activating the Wnt/β-catenin pathway and inhibiting osteogenic differentiation (Wang et al., 2017). These findings highlight let-7f as a key paracrine mediator between MSCs and tumor cells.

Immune microenvironment:Although direct evidence is limited, let-7f may modulate immune-related pathways. In follicular lymphoma, high let-7f expression correlates with better chemotherapy response, and immune-related signaling pathways (e.g., MAPK1, AKT1) are altered (Wang et al., 2011). In DLBCL, let-7f as an angiomiR may influence immune cell infiltration by regulating angiogenesis (Borges et al., 2016). These observations suggest a potential role for let-7f in tumor immunity that warrants further investigation.

Metabolic crosstalk: let-7f also influences metabolic interactions within the TME. For instance, paclitaxel-induced downregulation of let-7f in breast cancer increases TSP-1, inhibiting angiogenesis and thereby reducing oxygen/nutrient supply, which forces tumor cells to undergo metabolic reprogramming (Wei-Yang et al., 2015). This indirect effect links let-7f to TME-driven metabolic adaptation.

Let-7f mediates important crosstalk between tumor cells and stromal cells (e.g., via exosomal transfer from MSCs) and may influence immune cell function. These interactions further expand the role of let-7f in shaping the TME and its associated hallmarks.

Integrating the cancer hallmarks into the TME framework, let-7f functions as a multifaceted tumor suppressor that (1) regulates angiogenesis and metabolic reprogramming (Section 3.1), (2) suppresses invasion, metastasis, and stemness (Section 3.2), and (3) mediates crosstalk with stromal and immune cells (Section 3.3). By targeting a network of genes involved in ECM remodeling, EMT, stemness, glycolysis, and angiogenesis, let-7f counteracts key hallmarks of cancer within the TME. This integrated perspective eliminates redundancy and provides a clearer understanding of how let-7f exerts its anti-tumor effects in a microenvironment-dependent manner.

## 4 The relationship between let-7f and cancer therapy

### 4.1 Potential of let-7f as a therapeutic target in cancer

As a key member of the let-7 family, let-7f exhibits differential expression patterns in tumors and regulates critical signaling pathways that are closely linked to multiple cancer hallmarks, including sustained proliferation, evasion of apoptosis, activation of invasion and metastasis, and reprogrammed metabolism. These properties establish let-7f as a promising therapeutic target across various cancer types (Table 2).

**TABLE 2:** Key Targets and Molecular Mechanisms of let-7f.

Target	Function of target	Role in cancer	Related cancer types
HMGA2	small chromatin-associated proteins, architectural transcription factor	Let-7f downregulation → HMGA2 upregulation → promotion of tumor metastasis	Non-small cell lung cancer (Zafari et al., 2022) Neuroendocrine Neoplasms (Døssing et al., 2014)
ITGB1	oncoprotein	Let-7f downregulation →ITGB1 upregulation → promotion of tumor metastasis	Non-small cell lung cancer (Yang et al., 2021)
Periostin	matricellular protein	Let-7f targets the 3′UTR of periostin → reduces periostin expression → inhibits glioma cell growth, migration, and invasion	Glioma (Yan et al., 2015)
KLK10	A family of serine proteases	let-7f targets KLK10 → downregulation of KLK10 protein expression → reduced growth and proliferation of breast cancer cells	Ovarian cancer (White et al., 2010a), Renal cell carcinoma (White et al., 2010b)
TLR4/STAT3	transcriptional factor	Let-7f downregulates STAT3 → limits cell proliferation and enhances autophagy and apoptosis in glioma cells	Glioma (Yang et al., 2020b), Hepatocellular carcinoma (Li et al., 2022)
MYH9	the gene for non-muscle myosin heavy chain IIA	let-7f directly binds to the 3′UTR of its target MYH9 → inhibits gastric cancer invasion and metastasis	Gastric cancer (Liang et al., 2011)
CYP19A1	Cytochrome P450 Family 19 Subfamily A Member 1	let-7f directly inhibits aromatase gene expression → exerts tumor suppressive effects on breast cancer cells	Breast cancer (Shibahara et al., 2012)
ABCC5 ABCC10	Human ATP-binding cassette (ABC) transporters	Let-7f downregulation → negative regulation of MDR-related proteins → induction of ADR resistance	Acute myeloid leukemia (Cao et al., 2020)
TARBP2	double-stranded RNA binding protein	Hypoxia induces let-7f-5p/TARBP2 feedback loop → activates Wnt signaling pathway → regulates OS cell proliferation and invasion	Osteosarcoma (Chen et al., 2020)
HMGB1	chromatin-binding factor	SPARC expression → inhibition of miR-let-7f-1 expression → upregulation of HMGB1 → enhanced cisplatin sensitivity in SPARC-expressing cells	Medulloblastoma (Pannuru et al., 2014)

In nasopharyngeal carcinoma (NPC), miRNA sequencing of radiosensitive (CNE2) and radioresistant (HONE1) cells revealed that let-7f-5p is significantly downregulated in the resistant line. Its predicted targets include MAPK1 and SOS1, which are involved in the hallmark of resistance to cell death and DNA damage repair. This finding links let-7f to the regulation of treatment-specific cancer hallmarks and suggests its potential for optimizing radiotherapy targeting (Luo et al., 2022).

In uterine leiomyosarcoma (LMS), let-7f-5p is significantly lower in LMS tissues than in benign leiomyoma and correlates with patient prognosis. Functional studies show that let-7f-5p inhibits proliferation and migration by targeting CCND1 (cell cycle hallmark) and BCL2 (apoptosis evasion hallmark). Thus, exogenous let-7f-5p supplementation represents a potential therapeutic strategy that directly counteracts two core cancer hallmarks (de et al., 2025).

In non-muscle-invasive bladder cancer (NMIBC), low let-7f-5p expression is associated with tumor recurrence. Targeting the let-7f negative regulator Lin28 with the small molecule inhibitor C1632 upregulates let-7f-5p, thereby reducing cancer cell viability and migration. Notably, Lin28/let-7f axis is also linked to cancer stemness (a proposed enabling hallmark), offering a novel strategy to prevent recurrence (Shee et al., 2020).

In colorectal cancer (CRC), let-7f-5p functions as a tumor suppressor, and its downregulation is linked to CRC cell proliferation. The natural product rosmarinic acid antagonizes let-7f-5p′s inhibitory effect by modulating target genes AMER3 and SLC9A9, linking let-7f to metabolic and proliferative hallmarks. This interaction provides a new strategy for chemoprevention (Feng et al., 2021).

In breast cancer, let-7f inhibits estrogen synthesis by targeting the aromatase gene CYP19A1, directly interfering with the hallmark of growth signaling (hormone-dependent proliferation). Aromatase inhibitor (AI) treatment upregulates let-7f, forming a negative feedback loop (“AI–let-7f–aromatase”) that enhances therapeutic efficacy (Shibahara et al., 2012).

Importantly, TME modulation is a key aspect of let-7f′s therapeutic potential. In breast cancer, exosomal let-7f derived from human mesenchymal stem cells (hMSCs) is upregulated by SDF-1α or hypoxia—both common TME cues—and secreted via exosomes. Uptake of this exosomal let-7f by breast cancer cells inhibits proliferation and invasion, thereby counteracting hallmark capabilities. This finding supports the development of exosome-based delivery systems for let-7f (Egea et al., 2021) (Figure 3). In ovarian cancer, low let-7f expression associates with tumor progression, and its combination with miR-34a and miR-31 improves early diagnosis of epithelial ovarian cancer (EOC). This diagnostic role further underscores let-7f as an auxiliary therapeutic target (Kumar et al., 2022).

Notably, the therapeutic relevance of let-7f is multifaceted. In leukemia, let-7f downregulation induces doxorubicin (ADR) resistance by upregulating drug transporters ABCC5 and ABCC10 (linked to the hallmark of drug resistance, an emerging feature). Exogenous let-7f overexpression reverses this resistant phenotype (Cao et al., 2020). In triple-negative breast cancer (TNBC), high circulating let-7f levels are associated with reduced risk of chemotherapy-related cardiotoxicity, indicating its potential as a safety biomarker to optimize treatment selection (Zhu et al., 2018). Collectively, these studies demonstrate that let-7f functions across different cancer types by regulating multiple hallmarks—proliferation, apoptosis, drug resistance, and TME interactions. Therefore, developing let-7f as a therapeutic target requires individualized design based on the specific molecular and hallmark landscape of each tumor.

### 4.2 Impact of let-7f on cancer treatment efficacy

The expression level of let-7f is closely associated with the efficacy of cancer treatments, including chemotherapy, radiotherapy, and endocrine therapy. By modulating core cancer hallmarks (such as DNA damage repair, apoptosis, and angiogenesis) and TME components, let-7f directly influences treatment response.

In radiotherapy for nasopharyngeal carcinoma, let-7f-5p is significantly lower in radioresistant cells (HONE1) than in radiosensitive cells (CNE2). Its target genes MAPK1 and TP53 are involved in DNA damage repair and cell cycle regulation—key hallmarks determining radiosensitivity. Clinical validation shows that low let-7f-5p in tumor tissues is associated with increased local recurrence risk, supporting its role as a predictive biomarker for radiotherapy efficacy (Luo et al., 2022).

In chemotherapy, the impact of let-7f on efficacy varies by cancer type, reflecting context-dependent hallmark regulation. In colorectal cancer, let-7f-5p is higher in chemotherapy-resistant tissues and reduces chemosensitivity by inhibiting pro-apoptotic proteins p53 and Caspase-3 (apoptosis evasion hallmark) (Tie et al., 2018). In contrast, in leukemia, low let-7f promotes ADR resistance via ABCC5/ABCC10 (drug transport hallmark), and let-7f overexpression enhances ADR cytotoxicity, as confirmed *in vivo* (Cao et al., 2020). This bidirectional regulation indicates that let-7f′s role in chemotherapy efficacy is tumor-specific, likely due to tissue-specific expression of its target hallmarks.

In breast cancer treatment, let-7f′s influence on paclitaxel efficacy is particularly notable. Low-dose metronomic (LDM) paclitaxel downregulates let-7f, relieving suppression of THBS1 (encoding TSP-1, an anti-angiogenic factor). This enhances paclitaxel’s anti-angiogenic activity—a hallmark of sustained angiogenesis. Exogenous let-7f overexpression reverses this effect, diminishing LDM paclitaxel’s therapeutic outcome (Wei-Yang et al., 2015). This discovery reveals a non-cytotoxic mechanism linking let-7f to angiogenesis regulation and provides a basis for optimizing paclitaxel regimens.

In endocrine therapy, let-7f plays a crucial role in the efficacy of aromatase inhibitors (AIs) for estrogen receptor-positive (ER+) breast cancer. Let-7f directly targets CYP19A1 (aromatase gene), suppressing estrogen synthesis—a hallmark of growth signal addiction. AI treatment upregulates let-7f, and high let-7f expression correlates with prolonged disease-free survival, suggesting that let-7f potentiates AI efficacy (Shibahara et al., 2012). In uterine leiomyosarcoma, low let-7f-5p correlates with tumor aggressiveness, and exogenous let-7f-5p inhibits proliferation and invasion, indicating its potential as a complementary target for endocrine therapy (de et al., 2025).

In ovarian cancer, let-7f expression levels associate with response to carboplatin/paclitaxel. Although let-7f itself does not directly regulate chemosensitivity, its family member let-7d-3p (sharing overlapping targets in RAS and ErbB signaling) correlates with positive response. This suggests that let-7 family members, including let-7f, may influence chemotherapy efficacy through synergistic hallmark regulation (García-Vázquez et al., 2018).

In glioblastoma (GBM), let-7f expression correlates with overall survival (OS) in patients receiving combined radiotherapy and chemotherapy. High let-7f expression predicts significantly longer OS, indicating its role as a predictive biomarker for multimodal therapy efficacy (Lu et al., 2025).

Notably, let-7f also modulates treatment-related toxicities—an important clinical aspect linked to the TME and normal tissue hallmarks. In TNBC patients undergoing neoadjuvant chemotherapy (EC-D), high circulating let-7f is associated with reduced risk of cardiotoxicity, possibly via regulating cardiomyocyte apoptosis (e.g., inhibiting HAX-1 nuclear export) (Zhu et al., 2018). This finding provides a novel biomarker for assessing chemotherapy safety and indirectly improves treatment adherence and efficacy.

### 4.3 Relationship between let-7f and tumor therapy resistance

**FIGURE 4:**
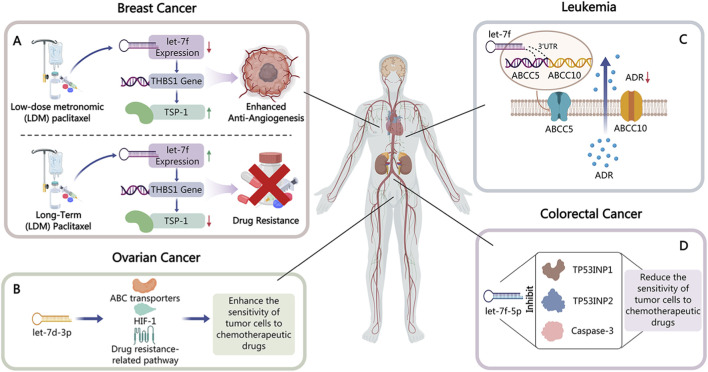
Regulatory roles of let-7f in cancer therapy response and drug resistance **(A)** In breast cancer, low-dose metronomic (LDM) paclitaxel treatment downregulates let-7f expression, leading to increased expression of the *THBS1* gene and subsequent upregulation of thrombospondin-1 (TSP-1). Elevated TSP-1 enhances anti-angiogenesis, contributing to the therapeutic effect. However, long-term LDM paclitaxel treatment results in an adaptive increase in let-7f expression, which suppresses *THBS1* expression and reduces TSP-1 levels. This loss of anti-angiogenic activity promotes drug resistance **(B)** Let-7d-3p enhances chemosensitivity in ovarian cancer by targeting drug resistance-related pathways. In ovarian cancer, let-7d-3p (a family member sharing high sequence homology with let-7f) suppresses the expression of ABC transporters and HIF-1, thereby inhibiting drug resistance-related signaling pathways. This regulation ultimately enhances the sensitivity of tumor cells to chemotherapeutic drugs **(C)** In leukemia, reduced let-7f expression leads to upregulation of the drug transporters ABCC5 and ABCC10 (by relieving let-7f-mediated suppression of their 3′-UTRs), which decreases intracellular accumulation of doxorubicin (ADR) and induces ADR resistance **(D)** In colorectal cancer, elevated let-7f-5p directly inhibits the expression of pro-apoptotic proteins including TP53INP1, TP53INP2, and Caspase-3, thereby reducing the sensitivity of tumor cells to chemotherapeutic drugs and promoting chemoresistance.

Let-7f plays a pivotal role in the development of tumor therapy resistance by regulating multiple hallmarks, including drug transport, apoptosis evasion, DNA damage repair, cancer stemness, and TME remodeling (Table 3; Figure 4).

**TABLE 3:** Major Functional Outcomes of let-7f.

Functional outcome	Mechanism of action	Related cancer types
Inhibit cell proliferation and induce apoptosis	Let-7f inhibits tumor proliferation by directly targeting multiple genes	NSCLC (Zafari et al., 2022) Glioma (Yan et al., 2015)
Inhibit invasion and metastasis	Regulate the expression of molecules related to extracellular matrix remodeling, epithelial-mesenchymal transition (EMT), cell migration and invasion	Gastric cancer (Liang et al., 2011) Glioma (Yan et al., 2015)
Sensitization chemotherapy and targeted therapy	Regulate drug transport, apoptosis pathway, DNA damage repair, tumor stem cells and microenvironment	Acute myeloid leukemia (Cao et al., 2020) Medulloblastoma (Pannuru et al., 2014)
Regulate tumor stem cells	High expression of let-7f can reduce the tumor stem cell-like population and decrease their sphere-forming ability and self-renewal capacity	Breast cancer (Egea et al., 2021)
Affect the tumor microenvironment	Regulate angiogenesis, stromal cell function, immune cell function and metabolism	Large B-Cell lymphoma (Borges et al., 2016)

In breast cancer paclitaxel resistance, let-7f regulates the anti-angiogenic factor TSP-1. LDM paclitaxel downregulates let-7f, relieving THBS1 suppression and enhancing anti-angiogenic activity. However, long-term treatment may adaptively increase let-7f, which then inhibits TSP-1, weakens anti-angiogenesis, and promotes resistance (a hallmark of sustained angiogenesis). Exogenous let-7f overexpression reduces LDM paclitaxel’s efficacy, while a let-7f inhibitor enhances it, revealing a non-canonical resistance mechanism (Wei-Yang et al., 2015) (Figure 4A).

In ovarian cancer, although let-7f itself is not directly implicated in carboplatin/paclitaxel resistance, let-7d-3p (a family member with high sequence homology) is associated with positive chemotherapy response. Let-7d-3p targets ABC transporters, HIF-1, and RAS pathways—hallmarks of drug resistance and hypoxic TME. Given the homology, let-7f may participate in similar resistance mechanisms (García-Vázquez et al., 2018). In uterine leiomyosarcoma, low let-7f-5p correlates with chemoresistance, and exogenous let-7f-5p enhances sensitivity to cisplatin and paclitaxel, likely via cell cycle and apoptosis regulation (de et al., 2025) (Figure 4B).

In leukemia, let-7f is significantly lower in the ADR-resistant cell line K562/A02 than in sensitive K562 cells, while its targets ABCC5 and ABCC10 (ABC transporters) are elevated. Let-7f directly binds to their 3′UTRs to inhibit expression, reducing drug efflux (a hallmark of multidrug resistance). *In vivo*, let-7f overexpression enhances ADR efficacy and reverses resistance (Cao et al., 2020) (Figure 4C).

In colorectal cancer, high let-7f-5p expression in chemotherapy-resistant tissues promotes resistance by directly inhibiting pro-apoptotic proteins (p53, TP53INP1, TP53INP2, Caspase-3), thereby suppressing chemotherapy-induced apoptosis—a hallmark of evasion of cell death. Downregulating let-7f-5p sensitizes CRC cells to 5-FU and oxaliplatin, offering a strategy to reverse resistance (Tie et al., 2018) (Figure 4D).

In glioblastoma, let-7f expression is lower in recurrent tumors than in primary ones and correlates with OS. Among patients receiving radiotherapy plus chemotherapy, high let-7f predicts longer OS, suggesting that let-7f may influence resistance by regulating DNA damage repair hallmarks independently of MGMT methylation (Lu et al., 2025).

Cancer stem cell (CSC)-mediated resistance is an emerging hallmark linked to let-7f. Let-7f inhibits CSC self-renewal and proliferation by targeting LIN28 and HMGA2. In NMIBC, high LIN28 correlates with low let-7f-5p, and the LIN28 inhibitor C1632 upregulates let-7f-5p, suppressing CSC activity and reducing recurrence (Shee et al., 2020). This demonstrates that let-7f can reverse therapy resistance by targeting the stemness hallmark.

Furthermore, TME dynamics play a critical role in let-7f-mediated resistance. In breast cancer, exosomal let-7f from hMSCs inhibits tumor cell proliferation and invasion. However, under prolonged chemotherapy pressure, tumor cells secrete SDF-1α, which downregulates let-7f in hMSCs, reduces exosomal let-7f secretion, and weakens tumor suppression—a mechanism illustrating how TME remodeling promotes resistance (Egea et al., 2021).

Collectively, let-7f contributes to tumor therapy resistance through multiple interconnected hallmarks and TME pathways: drug transport, apoptosis evasion, DNA damage repair, sustained angiogenesis, cancer stemness, and microenvironmental crosstalk. A deeper understanding of these context-specific mechanisms will provide a theoretical foundation for developing personalized strategies to overcome resistance.

## 5 Clinical applications and prospects of let-7f

### 5.1 Potential of let-7f as a tumor diagnostic biomarker

The development of reliable diagnostic biomarkers is essential for improving early cancer detection and patient prognosis. Traditional markers such as PSA and CEA often lack sufficient specificity and sensitivity. MicroRNAs, including let-7f, are stably present in body fluids, exhibit tissue-specific expression patterns, and are closely linked to tumorigenesis and progression, making them promising candidates for next-generation diagnostic biomarkers. This subsection summarizes the evidence supporting let-7f as a diagnostic biomarker across various cancer types.

In lung cancer, let-7f expression is closely related to diagnosis and prognosis. A study on lung adenocarcinoma used optimized Support Vector Regression (SVR) to identify let-7f-1 as one of 18 miRNA features significantly associated with patient survival time (Yerukala and Ho, 2017). In NSCLC, reduced let-7f levels in plasma exosomes correlated with tumor stage and could differentiate early from advanced patients, offering a non-invasive diagnostic approach (Silva et al., 2010). Moreover, serum let-7f levels correlated with TGF-β and VEGF, further supporting its diagnostic feasibility (Chaniad et al., 2020).

In colorectal cancer (CRC), multiple studies have demonstrated significant downregulation of let-7f-5p in plasma and stool. Notably, let-7f expression in stool showed high sensitivity and specificity for distinguishing CRC patients from healthy individuals (Ghanbari et al., 2015). Its expression also correlates with pathological features: higher let-7f levels are observed in well-differentiated, early-stage (I-II), and node-negative CRC, suggesting utility for early diagnosis (Yuan et al., 2019). Genetic polymorphism studies revealed that the AG/AA genotype at rs17276588 in the pri-let-7f flanking region reduces transcriptional activity and is associated with increased CRC risk (Yuan et al., 2019). Additionally, the rs10889677 polymorphism in the IL23R 3′UTR (a binding site for let-7e/let-7f) shows that the AA genotype increases CRC risk, while the CC genotype is protective (Mosallaei et al., 2019).

In prostate cancer (PCa), where PSA often fails to distinguish PCa from benign prostatic hyperplasia (BPH), plasma let-7f-5p was significantly upregulated in PCa patients. Combining let-7f-5p with PSA yielded superior diagnostic performance (AUC = 0.904) compared to PSA alone (0.795) or let-7f-5p alone (0.782), indicating that let-7f serves as a complementary biomarker (Ge et al., 2020).

In gynecological tumors, let-7f is downregulated in uterine leiomyosarcoma (LMS) tissues, especially in elderly patients, providing a potential diagnostic reference (de et al., 2019). In ovarian cancer, reduced plasma let-7f combined with miR-205 improved diagnostic accuracy, particularly for early-stage disease (Zheng et al., 2013). Furthermore, highly invasive SKOV-3ip cells expressed significantly lower let-7f than less invasive SKOV-3 cells, linking let-7f to invasion and malignancy assessment (Liang et al., 2010).

In other tumor types, let-7f also shows diagnostic potential. In gastric cancer, let-7f is downregulated in tumor tissues and in high-metastasis cell lines, with levels negatively correlating with metastatic capacity (Liang et al., 2011). In GBM, let-7f expression changes upon recurrence and correlates with treatment regimens, suggesting utility for monitoring recurrence (Lu et al., 2025). In pancreatic cancer, decreased let-7f in fine-needle aspiration specimens can be combined with other miRNAs for molecular diagnosis (Ali et al., 2012). In papillary thyroid carcinoma, reduced let-7f expression is associated with tumor aggressiveness and may aid prognosis assessment (Geraldo et al., 2012).

Let-7f is a stable, non-invasive, and tissue-associated diagnostic biomarker across multiple cancers. Its expression correlates with tumor stage, differentiation, and metastasis, and it can complement existing markers such as PSA. However, current studies face limitations in standardization and sample size; future multicenter large-scale trials are needed to validate its clinical utility.

### 5.2 Exploratory applications of let-7f in cancer therapy

The therapeutic exploration of let-7f focuses on three main areas: (1) as a direct therapeutic target, (2) as a chemosensitizer, and (3) as an exosome-mediated delivery agent. Its tumor-suppressive functions provide the biological rationale for these applications.

As a direct therapeutic target, let-7f exerts anti-tumor effects by regulating key target genes. In gastric cancer, let-7f targets MYH9 to inhibit invasion and migration (Liang et al., 2011). In glioblastoma, it targets periostin to suppress proliferation, migration, and invasion (Yan et al., 2015). In NSCLC, let-7f targets HMGA2, ARID3B, SMARCAD1, and FZD3 to inhibit tumor cell proliferation (Zafari et al., 2022). These findings suggest that exogenous let-7f mimics could serve as a novel targeted therapy.

As a chemosensitizer, let-7f enhances sensitivity to chemotherapy. In leukemia, the multidrug-resistant cell line K562/A02 has low let-7f expression, leading to upregulation of ABCC5/ABCC10. Transfection with let-7f mimics reduces these transporters and restores ADR sensitivity (Cao et al., 2020). In breast cancer, low-dose metronomic (LDM) paclitaxel downregulates let-7f, which in turn upregulates the anti-angiogenic factor TSP-1. Overexpression of let-7f inhibits this TSP-1 upregulation, indicating that let-7f modulates chemosensitivity via angiogenesis regulation (Tao et al., 2015). In ovarian cancer, let-7f inhibits proliferation by targeting KLK10, and its expression correlates with chemotherapy response (White N. et al., 2010).

Exosome-mediated delivery represents a promising direction. Under SDF-1α or hypoxic stimulation, human mesenchymal stem cells (hMSCs) upregulate let-7f and secrete it via exosomes. These exosomal let-7f are taken up by breast cancer cells (4T1), inhibiting proliferation and invasion, and suppressing tumor growth *in vivo* (Egea et al., 2021) (Figure 3). This provides an experimental basis for using exosomes as delivery vehicles to overcome stability and targeting challenges.

In hormone therapy, let-7f plays a role in aromatase inhibitor (AI) efficacy. In ER + breast cancer, AIs such as letrozole upregulate let-7f, which then directly targets CYP19A1 (aromatase) to inhibit its expression, thereby enhancing AI efficacy (Shibahara et al., 2012). In endometrial stromal cells from endometriosis patients, AI treatment increases let-7f and inhibits cell migration (Cho et al., 2016).

Preclinical studies demonstrate that let-7f can act as a therapeutic target, a chemosensitizer, and a payload for exosome-based delivery. However, these applications are still at the experimental stage. Future work should focus on optimizing delivery systems, improving *in vivo* stability and targeting, and validating efficacy and safety in clinical trials. Combination strategies with chemotherapy, radiotherapy, or immunotherapy are also worth exploring.

### 5.3 Challenges and prospects for clinical translation

Despite the promising preclinical evidence, the clinical translation of let-7f faces several challenges, along with clear opportunities for future development.

#### 5.3.1 Challenges

1. Standardization of detection methods. Most studies use qRT-PCR or microarrays, but differences in sample processing, RNA extraction, reverse transcription primers, and reference genes lead to poor comparability. For example, plasma let-7f detection methods vary widely across studies (Ghanbari et al., 2015; Silva et al., 2010; Cho et al., 2016). Moreover, different isoforms (let-7f-5p vs. let-7f-3p) may have distinct functions, yet most studies do not distinguish them (Zafari et al., 2022; Cao et al., 2020). Establishing standardized protocols is a prerequisite for clinical use.
2. Specificity and sensitivity limitations. Although let-7f is dysregulated in many cancers, it is not cancer-specific. Altered let-7f levels are also found in non-cancerous conditions such as abdominal aortic aneurysm (Spear et al., 2019), myocardial infarction (Chen-Yun et al., 2019), and diabetic nephropathy. Patient age, gender, and treatment history further influence its expression (de et al., 2019; Ge et al., 2014). Combining let-7f with other biomarkers into integrated diagnostic panels may improve specificity and sensitivity.
3. Delivery and targeting challenges in therapy. Exogenous let-7f mimics are prone to nuclease degradation and lack tumor specificity. While exosome- and liposome-based delivery systems have shown promise, their manufacturing, targeting efficiency, and biosafety need further optimization. Additionally, let-7f has cell-type-dependent effects (e.g., it protects vascular endothelial cells (Zhao et al., 2021) but suppresses tumor cells (Liang et al., 2011; Yan et al., 2015)), raising the risk of off-target effects. More precise delivery systems are required.
4. Limitations in clinical studies. Most current studies have small sample sizes (e.g., tens to a hundred cases in CRC diagnostic studies (Ghanbari et al., 2015; Yuan et al., 2019)) and are retrospective. Prospective, multicenter trials are lacking. Furthermore, prognostic associations are inconsistent across studies: in LMS, let-7f correlated with age but not with overall survival (de et al., 2019), whereas in ovarian cancer, low let-7f was associated with poor prognosis (Zheng et al., 2013). Large-scale, well-designed studies are needed to resolve these discrepancies.

#### 5.3.2 Future prospects

1. Multi-omics integration and AI-assisted diagnosis. Integrating let-7f with other miRNAs, genes, and protein biomarkers using machine learning algorithms can improve diagnostic and prognostic models (Yerukala and Ho, 2017). AI can also help analyze clinicopathological associations to support clinical decision-making.
2. Development of novel delivery systems. Tumor microenvironment-responsive nanocarriers (e.g., polymeric nanoparticles, lipid nanoparticles, engineered exosomes) could achieve targeted let-7f delivery with reduced side effects (Egea et al., 2021). Gene-editing tools such as CRISPR/Cas9 may also be employed to regulate let-7f expression.
3. Combination therapeutic strategies. Combining let-7f-based approaches with conventional chemotherapy, radiotherapy, or immunotherapy may enhance treatment outcomes. For example, let-7f mimics combined with chemotherapeutic agents can improve chemosensitivity (Cao et al., 2020; Tao et al., 2015). Let-7f may also modulate immune cells in the TME to potentiate immunotherapy.
4. Expansion to other diseases. Preliminary evidence of let-7f′s roles in abdominal aortic aneurysm (Spear et al., 2019), myocardial infarction (Chen-Yun et al., 2019), and diabetic nephropathy suggests broader diagnostic and therapeutic applications. It may also serve as a reference gene in bone aging and senescence studies (Kaur et al., 2022).
5. Deeper mechanistic research. Further investigation is needed into let-7f′s regulatory roles within the TME, such as its effect on tumor-associated macrophage polarization (Allela et al., 2025) and its interaction with the TGF-β/ALK5 pathway in angiogenesis (Dhahri et al., 2017). Elucidating these mechanisms will strengthen the theoretical foundation for clinical translation.

Collectively, let-7f holds substantial promise as a diagnostic biomarker (stable, non-invasive, and complementary to existing markers) and as a therapeutic agent (direct targeting, chemosensitization, and exosome-mediated delivery). However, several challenges remain: standardization of detection methods, specificity improvement, delivery system optimization, and validation through large-scale prospective clinical trials. Future interdisciplinary efforts—integrating multi-omics, AI, novel nanocarriers, and combination therapies—will be essential to advance the clinical translation of let-7f. Moreover, its potential applications in non-cancer diseases warrant further exploration. With continued research, let-7f may become a valuable tool for precision diagnosis and treatment not only in oncology but also in other human diseases.

## 6 Conclusion

This study systematically explores the role and significance of let-7f in tumorigenesis and progression. Through a comprehensive analysis of relevant literature, it reveals the expression characteristics, regulatory mechanisms, and key functions of let-7f in the biological behavior of different tumor types. The findings indicate that let-7f, as an important tumor-suppressive miRNA, exhibits dysregulated expression closely associated with the development and progression of various cancers, highlighting its potential value as both a diagnostic biomarker and a therapeutic target.

Regarding differential expression in tumors, let-7f shows a downregulation trend in many cancers, including colorectal (Yuan et al., 2019), gastric (Liang et al., 2011), glioblastoma (Yan et al., 2015), breast (Egea et al., 2021), and ovarian (Zheng et al., 2013) cancers. This downregulation can be induced by various mechanisms such as genetic polymorphisms (e.g., rs17276588) (Yuan et al., 2019), lncRNA regulation (e.g., by FAM222A-AS1) (Song et al., 2022), and DNA methylation (Kumar et al., 2022). Furthermore, let-7f expression levels correlate closely with tumor pathological features, such as tumor size (Ge et al., 2014), clinical stage (Yuan et al., 2019), and metastatic potential (Liang et al., 2011), suggesting its potential as a biomarker for tumor progression.

Concerning its mechanisms in tumorigenesis and progression, let-7f exerts tumor-suppressive effects by targeting multiple oncogenes and signaling pathways. For example, in colorectal cancer, let-7f inhibits tumor cell invasion and metastasis by targeting MYH9 (Song et al., 2022); in glioblastoma, let-7f targets periostin to suppress tumor cell proliferation, migration, and vascular mimicry (Yan et al., 2015; Xue et al., 2016); in gastric cancer, let-7f inhibits metastasis via targeting MYH9 (Liang et al., 2011); and in lung cancer, let-7f inhibits cell proliferation by targeting genes like HMGA2 and FZD3 (Zafari et al., 2022). Additionally, let-7f participates in regulating the tumor microenvironment, such as influencing tumor growth via exosomal release (Egea et al., 2021), and plays a role in tumor metabolic reprogramming (Tao et al., 2015).

In the context of cancer therapy, let-7f is closely linked to treatment efficacy and resistance. For instance, in leukemia, downregulation of let-7f is associated with doxorubicin resistance, while its overexpression can increase cellular sensitivity to the drug (Cao et al., 2020); in breast cancer, low-dose metronomic paclitaxel therapy downregulates let-7f to upregulate TSP-1, exerting an anti-angiogenic effect (Tao et al., 2015); and in ovarian cancer, let-7f expression correlates with chemotherapy response (García-Vázquez et al., 2018). Moreover, let-7f itself holds promise as a therapeutic target, with strategies aimed at restoring its expression or mimicking its function to inhibit tumor growth.

Regarding clinical application prospects, let-7f demonstrates potential as a diagnostic biomarker for tumors. For example, in colorectal cancer, let-7f expression levels in plasma and stool can serve as markers for early diagnosis (Ghanbari et al., 2015); in liver cancer, serum let-7f expression correlates with tumor size and recurrence (Ge et al., 2014); and in ovarian cancer, combined detection of serum let-7f and miR-205 improves diagnostic accuracy (Zheng et al., 2013). However, the clinical translation of let-7f still faces numerous challenges, including the standardization of detection methods, the influence of individual variation, and the optimization of *in vivo* delivery systems.

In conclusion, let-7f plays a significant role in tumorigenesis and progression. Its aberrant expression and functional dysregulation are closely associated with the occurrence, development, and prognosis of various cancers. In-depth research into the regulatory mechanisms and targets of let-7f will provide new insights and strategies for cancer diagnosis and treatment. Future studies should further explore the specific mechanisms of let-7f in different cancers, develop more sensitive and specific detection methods, and design effective therapeutic regimens to advance the translation of let-7f from basic research to clinical application.

## Glossary

AAA: Abdominal aortic aneurysm
ADR: Doxorubicin
AngiomiR: Pro-angiogenic miRNA
ADAMTS1: A Disintegrin and Metalloproteinase with Thrombospondin Motifs 1
AI: Aromatase inhibitor
AI: Artificial intelligence
β2-AR: β2-adrenergic receptor
BC-NEFs: Breast cancer, patients with neuroendocrine features
BPH: Benign prostatic hyperplasia
CCND1: cyclin D1
ccRCC: Clear cell RCC
CEA: Carcinoembryonic antigen
C/EBP: CCAAT/enhancer-binding protein β
CeRNA: Competing endogenous RNA
CRC: Colorectal cancer
CSC: Cancer stem cell
DFS: Disease-free survival
DLBCL: Diffuse large B-cell lymphoma
ECs: Extracellular condensates
EC-D: Epirubicin/cyclophosphamide followed by docetaxel
EMT: epithelial-mesenchymal transition
ER+: Estrogen receptor-positive
EVs: Extracellular vesicles
EOC: Epithelial ovarian cancer
FASN: Fatty acid synthase
5-FU: 5-fluorouracil
FNA: Fine-needle aspiration
FZD3: Frizzled Class Receptor 3
PFKFB3: 6-phosphofructo-2-kinase/fructose-2,6-biphosphatase 3
GBM: glioblastoma
HCC: Hepatocellular carcinoma
hMSCs: Human bone marrow-derived mesenchymal stem cells
HMGA2: High Mobility Group AT-Hook 2
HK2: Hexokinase 2
*H. pylori*: *Helicobacter pylori*
MDR: Multidrug resistance
MI: Myocardial infarction
MMP-9: Matrix metalloproteinase-9
MYH9: Myosin Heavy Chain 9
MVD: Microvessel density
NEFs: Neuroendocrine features
IGF2BP1: Insulin-like Growth Factor 2 mRNA-Binding Protein 1
IDC: Invasive ductal carcinoma
LDM: Low-dose metronomic
LM: Leiomyoma
LMS: Leiomyosarcoma
LncRNAs: Long non-coding RNAs
IDC: Invasive ductal carcinoma
IGF2BP1: Insulin-like Growth Factor 2 mRNA-Binding Protein 1
MDR: Multidrug resistance
miRNAs: MicroRNAs
MMP-9: Metalloproteinase-9
MSCs: Mesenchymal stem cells
MVD: Microvessel density
NSCLC: Non-small cell lung cancer
NMIBC: Non-muscle-invasive bladder cancer
ncRNAs: Non-coding RNAs
OGD/R: Oxygen-glucose deprivation/reoxygenation
OS: Overall survival
PAD: Peripheral artery disease
PCa: Prostate cancer
PCNSL: Primary central nervous system lymphoma
PFKFB3: 6-phosphofructo-2-kinase/fructose-2,6-biphosphatase 3
POSTN: Periostin
Pre-let-7f: Precursor of let-7f
PTC: Papillary thyroid carcinoma
PSA: Prostate-specific antigen
RCC: Renal cell carcinoma
qRT-PCR: Real-time polymerase chain reaction
SDF-1α: Stromal cell-derived factor 1α
SREBP1: Sterol regulatory element-binding protein 1
SNAI1: Snail Family Transcriptional Repressor 1
SIV: Simian immunodeficiency virus
SVR: Support Vector Regression
TAMs: Tumor-associated macrophages
TARBP2: TAR RNA-Binding Protein 2
TGF-β: Transforming growth factor-beta
TIMP-1: Tissue inhibitor of metalloproteinases-1
TME: tumor microenvironment
TNBC: Triple-negative breast cancer
TP53INP1: p53-induced nuclear protein 1
TSP-1: Thrombospondin-1
3′-UTR: 3'-untranslated region
VM: Vascular mimicry
VEGF: Vascular endothelial growth factor
WHO: World Health Organization
